# Simulation via instant messaging-Birmingham advance (SIMBA) model helped improve clinicians’ confidence to manage cases in diabetes and endocrinology

**DOI:** 10.1186/s12909-020-02190-6

**Published:** 2020-08-18

**Authors:** Eka Melson, Meri Davitadze, Manal Aftab, Cai Ying Ng, Emma Ooi, Parisha Blaggan, Wentin Chen, Thia Hanania, Lucretia Thomas, Dengyi Zhou, Joht Singh Chandan, Latha Senthil, Wiebke Arlt, Sailesh Sankar, John Ayuk, Muhammad Ali Karamat, Punith Kempegowda

**Affiliations:** 1 Institute of Metabolism and Systems Research, University of Birmingham, Birmingham, UK; 2 University Hospitals Birmingham NHS Foundation Trust, Birmingham, UK; 3 Clinic “Cardio”, Tbilisi, Georgia; 4 Georgian-American Family Medicine Clinic “Medical House”, Tbilisi, Georgia; 5 RCSI & UCD Malaysia Campus, George Town, Malaysia; 6 University of Birmingham Medical School, Birmingham, UK; 7 Institute of Applied Health Research, University of Birmingham, Birmingham, UK; 8 Diabetes and Endocrinology Specialist Training Committee, Health Education West Midlands, Birmingham, UK

## Abstract

**Background:**

Simulation-based learning (SBL) has been increasingly used in both undergraduate and postgraduate medical training curricula. The aim of Simulation via Instant Messaging-Birmingham Advance (SIMBA) is to create a simple virtual learning environment to improve trainees’ self-reported confidence in diabetes and Endocrinology.

**Methods:**

This study was done as part of the continuous professional development for Health Education England West Midlands speciality trainees in diabetes and Endocrinology. Standardized transcripts of anonymized real-life endocrinology (endocrine session) and diabetes cases (diabetes session) were used in the simulation model. Trainees interacted with moderators through WhatsApp® in this model. All cases were then discussed in detail by a consultant endocrinologist with reference to local, national and international guidelines. Trainee acceptance rate and improvement in their self-reported confidence levels post-simulation were assessed.

**Results:**

70.8% (*n* = 17/24) and 75% (*n* = 18/24) strongly agreed the simulation session accommodated their personal learning style and the session was engaging. 66.7% (*n* = 16/24) strongly felt that the simulation was worth their time. In the endocrine session, there was a significant improvement in trainees’ confidence in the management of craniopharyngioma (*p* = 0.0179) and acromegaly (*p* = 0.0025). There was a trend towards improved confidence levels to manage Cushing’s disease and macroprolactinoma. In diabetes session, there was a significant improvement in trainees’ confidence to interpret continuous glucose monitor readings (*p* = 0.01). There was a trend towards improvement for managing monogenic diabetes, hypoglycaemic unawareness and interpreting Libre readings. Overall, there was a significant improvement in trainees’ confidence in managing cases that were discussed post-simulation.

**Conclusion:**

SIMBA is an effective learning model to improve trainees’ confidence to manage various diabetes and endocrine case scenarios. More sessions with a variety of other speciality case scenarios are needed to further assess SIMBA’s effectiveness and application in other areas of medical training.

## Background

The field of medical education has been constantly evolving over the years with the utilization of relatively newer learning methods such as problem-based learning (PBL) and simulation-based learning (SBL). SBL has also been proven to be a superior learning method compared to both LBL and PBL [1, 2]. Despite the increasing popularity of SBL and PBL, lecture-based learning (LBL) is still the most widely used teaching method in both undergraduate and postgraduate medicine [3]. Evidence from research has shown that LBL requires the least amount of resources compared to other learning methods. Studies have shown that students who undertake more LBL mostly memorize the facts taught and have lower levels of knowledge retention and application [4]. Despite attempts to revise the curriculum, questions remain about how well the current model of lectures translates into the clinical environment [5].

Simulation-based medical education is diverse with many options such as medically stable patients, live actors, hybrid simulation, social media-based teaching which includes Facebook, WhatsApp and Twitter, or computer-based simulators [6–9]. In general, simulation is an educational activity that utilizes any or a combination of the above simulative tools with the purpose to imitate real-life clinical situations [10]. The goal of such simulation is to replicate real-life scenarios for the purpose of learning with feedbacks and assessments without putting patients at risk [11].

SBL has been widely used across disciplines in medicine especially in the more interventional specialities such as anaesthesiology [12, 13], emergency medicine [14], cardiology [15] and radiology [16, 17]. To our knowledge, SBL has not been used in the field of endocrinology except for the use of SimMan 3G manikin for the management of endocrine emergencies [18]. Although the use of SimMan in training provides trainees with realistic experiences inpatient management, they are expensive and not widely available [19]. Furthermore, even though the management of clinical emergencies is important, most cases in endocrinology are managed in an outpatient/clinic setting and hence endocrine emergencies simulation would not be representative of the training in this speciality. Therefore, there is a need for a minimal-cost SBL model with a focus on day-to-day cases in Diabetes and Endocrinology.

## Aim

The aim of SIMBA was to create a minimal cost simulation environment based on real-life situations to improve trainees’ confidence to manage a variety of case scenarios in Diabetes and Endocrinology.

## Methods

This study was conducted in July 2019 (endocrine session – pituitary case scenarios) and October 2019 (diabetes session – diabetes case scenarios), as part of a continuous professional and educational development for clinicians-in-training in Diabetes and Endocrinology in Health Education West Midlands (HEWM) deanery. All specialist trainee registrars specializing in Endocrinology and Diabetes or Metabolic medicine participated in the study.

**Fig. 1:**
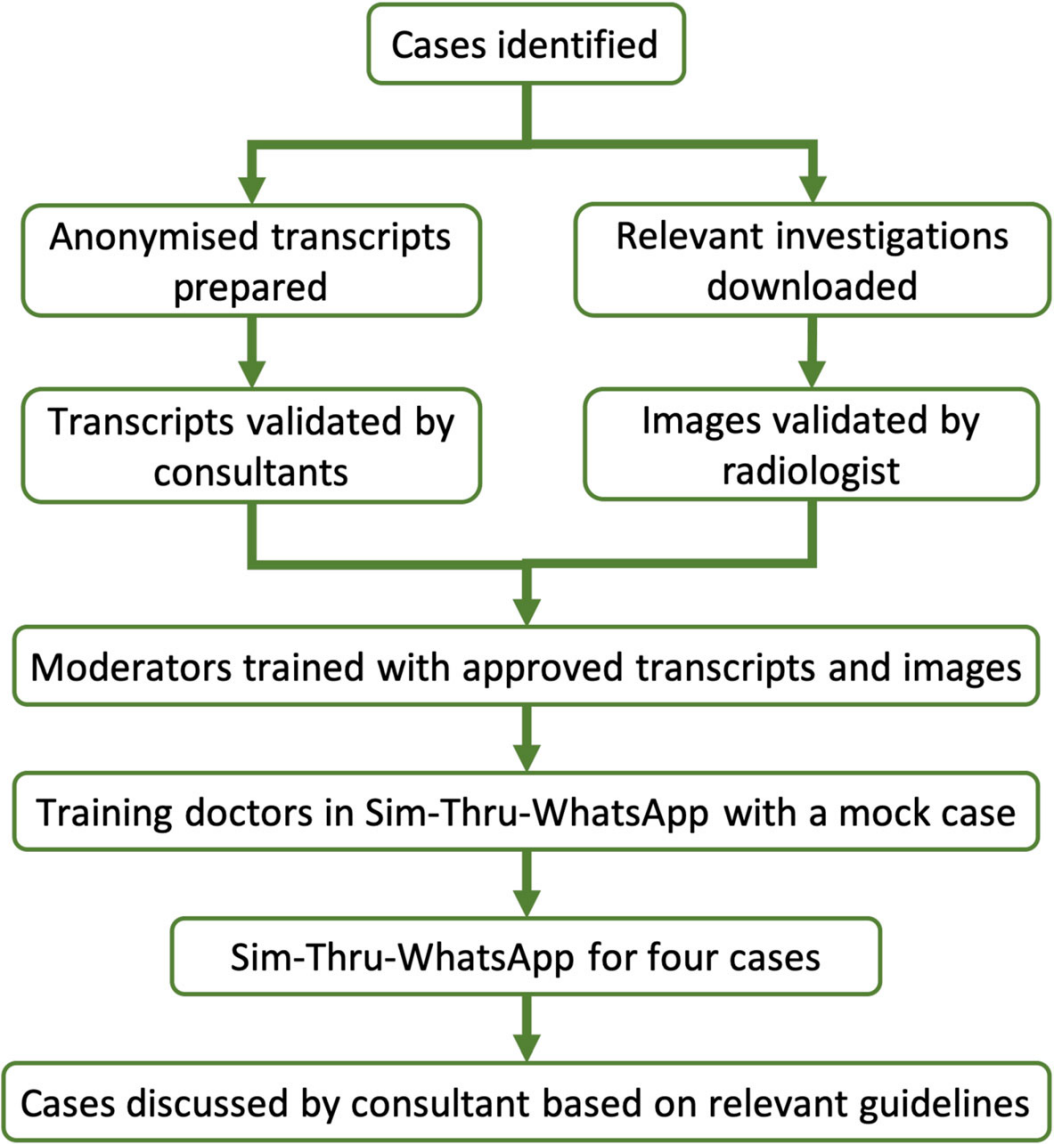
SIMBA protocol

SIMBA was based on interactive SBL through WhatsApp®. We initially identified five real-life case scenarios for endocrine and diabetes sessions. Following approval from specialists, anonymised transcripts were created on these case scenarios. These transcripts included medical history, clinical examinations, investigation results, imaging and other relevant information that would enable trainees to diagnose the case, propose management and follow-up plans. No patient identifiable data was included in the transcript. These transcripts were validated and approved by a consultant endocrinologist with specialist expertise to ensure that they portray real-life scenarios of respective cases. While the images used in endocrine session were approved by a consultant neuro-radiologist with a special interest in pituitary pathology, similar approval was obtained for continuous glucose monitoring and Libre readings for diabetes session (Fig. 1).

For the endocrine session, standardized transcripts of five anonymized pituitary cases — Non-Functioning Pituitary Adenoma (NFPA), craniopharyngioma, macroprolactinoma, acromegaly and Cushing’s disease — were prepared. For diabetes session, standardized transcripts of four anonymized diabetes cases — interpreting Libre readings, interpreting continuous glucose monitor (CGM) readings, hypoglycaemic unawareness, and monogenic diabetes — were created. Along with the simulated case scenarios, non-simulated case scenarios were identified by the consultant specialist supervising the specific session based on their prevalence and relevance to daily clinical practice. Non-simulated cases were different from simulated scenarios but matched in frequency, exposure and challenge to simulated ones in real-world practice for participants. This was to match simulated and non-simulated scenarios as much as possible so that SIMBA was the only variant between the two. The idea behind the analysis comparing simulated and non-simulated cases was for the participant to draw their experience from day-to-day practices while reporting their confidence to manage such case pre- and post-SIMBA session. For example, in diabetes session, Libre and CGM were compared with blood glucose meters and ketone meters; hypoglycaemic unawareness was compared with neuropathy and monogenic diabetes was compared with gestational diabetes.

In the endocrine session, five moderators were chosen to participate in the study, and the number of moderators was increased to ten for diabetes session based on the feedback from the endocrine session. The moderators were recruited considering their interest in the field of endocrinology and their motivation to participate in the innovative method of learning. To ensure their proficiency, the selected moderators were provided with the finalized transcripts 3 weeks prior to the session. The moderators familiarized themselves with the transcripts followed by at least five mock simulation sessions amongst each other. In order to ensure there was no heterogeneity in the responses, these moderators were then tested by the senior authors of the study. The role of the moderators was to simulate a patient, a senior clinician and a multi-disciplinary team (MDT) liaison at different points of the simulation. At the start of the simulation, the moderator took up the role of a patient from whom trainees requested a history of presenting complaints and relevant associated medical history. Where the trainees requested physical, biochemical, radiological or any other relevant examination results, moderators simulated a senior clinician to provide this information. Lastly, the moderators played the role of MDT liaison when the trainee combined all relevant information to arrive at the diagnosis, management and follow-up plans. Moderators were instructed to give the relevant information that is provided in the transcripts. If they were asked for information not provided in the transcript, they would reply saying “this information is not available”.

**Fig. 2:**
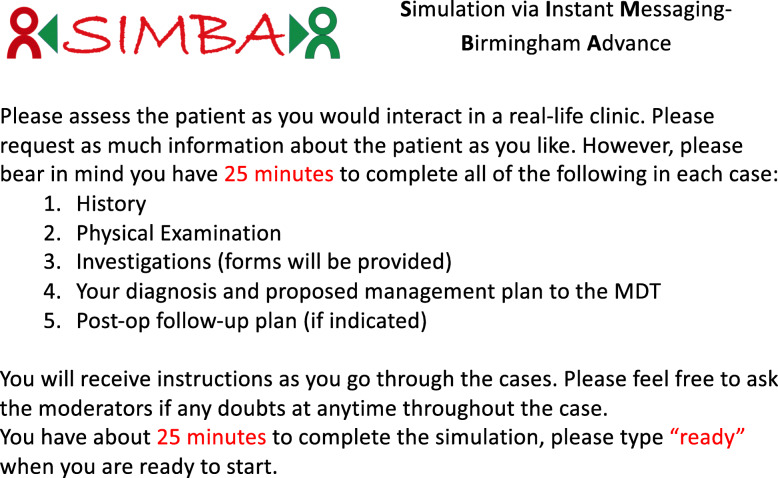
Instructions provided pre-simulation

On the day of the simulation, each moderator was assigned to a small group of trainees (two to four) with whom they would be replying in parallel. All trainees had been asked in advance to bring their own computers/laptops/notebooks through which they connected with the moderators via WhatsApp® Web application. The session started with the information in Fig. 2 via WhatsApp®. In summary, the trainees were instructed to approach the cases as they would in their daily clinical practice. Once the trainees were ready, the simulation was initiated by providing them with the presenting complaint of the patient. At this point, the moderator played the role of a patient answering relevant questions from the trainees to provide medical history. Once the history taking was complete, the moderators took up the role of a senior clinician providing necessary and relevant examination findings when asked by the trainee. The moderator prompted the trainee when they completed history and examination advising them to move to the next step of evaluation. If the trainee then proceeded to request blood tests, they were sent an electronic blood tests form to request the necessary investigations. Once the completed form was returned via WhatsApp, the moderators replied with the results of the requested blood test. If the trainee proceeded for a dynamic function test and/or radiologic investigation, the process for request and provision of results as for blood investigations was repeated. Once these steps were complete, the moderator prompted the trainee to summarise the findings and propose the diagnosis and management plan to MDT. As these cases had been discussed in MDT in real-life, the moderator compared the trainee’s reply to the factual outcome. Should they match, the moderator informed the same to the trainee. If they did not match, the moderator advised the trainee with the correct diagnosis. In both scenarios, the moderator asked the trainee for a follow-up plan. Once the trainees provided follow-up plans, the simulation ended. If at any point during simulation, a trainee requested information that was unavailable on the transcript (e.g. ordering an inappropriate investigation or skipping a crucial step in diagnosis/management), they were prompted by the moderators that the information was not available or was appropriately guided back to the relevant step.

To allow participants to familiarize themselves to the simulation model, the first case in each session was run as a trial. After the first case, the case and its approach were discussed in detail in line with current guidelines by an expert, which further helped the trainees to better understand the course of the simulation session. We include an example WhatsApp transcript as a supplement to the paper for a better understanding of the model.

During the endocrine session, the case scenario of non-functioning pituitary adenoma (NFPA) was run as a trial. Following this, the trainees underwent simulation case scenarios for macroprolactinoma, craniopharyngioma, acromegaly and Cushing’s disease, followed by respective case discussions with consultant endocrinologist.

During the diabetes session, the case scenario of interpreting Libre readings was chosen as a trial similar to NFPA in endocrine session. This was followed by case scenarios for interpreting CGM readings, hypoglycaemic unawareness, and monogenic diabetes, followed by case discussions with relevant approaches in detail.

During post-simulation discussions, the consultant focused on the appropriate approach to the cases, in relation to the evidence-based international, national and local hospital guidelines as appropriate, in that order of hierarchy, for each specific condition [20–26]. Structured feedback/debriefing occurred at the end of the session when the chair discussed the cases and highlighted the lessons learned by getting the trainees to reflect on their performance and discuss strategies for using these lessons to improve their daily practice. The discussions were interactive, and the trainees had ample opportunity to ask any further questions regarding the diagnosis or management of the simulated cases. The trainees were not ranked or scored on their performance. However, they were informed about the accuracy of their diagnosis during the simulations by the moderators as described above.

The confidence of the trainees (measured using a Likert scale ranging from strongly disagree to strongly agree) in approaching different pituitary and diabetes cases was assessed pre- and post-simulation [27, 28]. These data were then categorised into three groups: (i) confident: for those who responded with strongly agree and agree; (ii) not confident: for those who responded with disagree and strongly disagree; (iii) unsure: for those who responded with agree somewhat, disagree somewhat and undecided. The confidence levels of managing cases pre- and post-simulation are reported using frequencies, percentages, and are displayed in bar charts. Due to the nature of the data, Wilcoxon rank-sum tests (significance set at *p* < 0.05) were deemed appropriate (using STATA MP/4 (Statacorp 2017)) to statistically compare confidence levels pre- and post-simulation. Significant tests are highlighted using an asterisk.

Improvements in trainees’ confidence levels pre- and post-simulation of simulated scenarios (endocrine session – NFPA, craniopharyngioma, macroprolactinoma, acromegaly, Cushing’s disease; diabetes session - interpreting Libre reading, interpreting CGM reading, hypoglycaemic unawareness, and monogenic diabetes) vs. non-simulated scenarios (endocrine session – microprolactinoma, pituitary apoplexy, thyrotropinoma, gonadotropinoma, pituitary carcinoma; diabetes session - neuropathy, gestational diabetes, blood glucose meters, and ketone meters) were also displayed using frequencies, percentages, bar charts, and were also statistically tested using Wilcoxon rank-sum.

In addition to views on the management of the cases, trainees were also asked to comment on their overall impression of the session, the consultant’s contribution during the discussion and their interaction with the moderators.

## Results

### Trainee satisfaction and confidence

In the endocrine session, 70.8% (*n* = 17/24) strongly agreed and 29.2% (*n* = 7/24) agreed that SIMBA was successful, and it accommodated their personal learning style. 75% (*n* = 18/24) strongly agreed and 25% (*n* = 6/24) agreed that it was engaging. 66.7% (*n* = 16/24) strongly felt that the simulation was worth their time and 33.3% (*n* = 8/24) agreed to this.

**Fig. 3:**
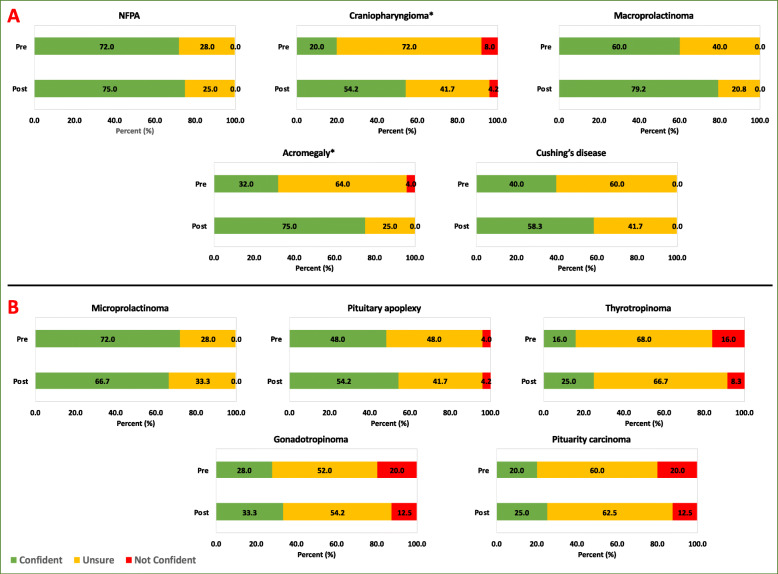
Illustration of changes in confidence levels for managing simulated (**a**) and non-simulated endocrine cases (**b**). *p < 0.05

There was a significant improvement in trainees’ self-reported confidence levels for the management of craniopharyngioma (*p* = 0.0179) and acromegaly (*p* = 0.0025). There was a trend towards improved confidence levels to other simulated endocrine cases; macroprolactinoma (*p* = 0.1498), and Cushing’s disease (*p* = 0.2040) (Fig. 3A). We did not see such trend when trainees were assessed for their confidence to manage non-simulated pituitary cases; pituitary carcinoma (*p* = 0.9335), microprolactinoma (p = 0.1498), pituitary apoplexy (*p* = 0.6913), gonadotropinoma (*p* = 0.3705) and thyrotropinoma (*p* = 0.3100) (Fig. 3B).

Overall, there was a significant improvement in trainee’s confidence in managing simulated pituitary cases (*p* = 0.0002) compared to non-simulated cases (*p* = 0.0655).

**Fig. 4:**
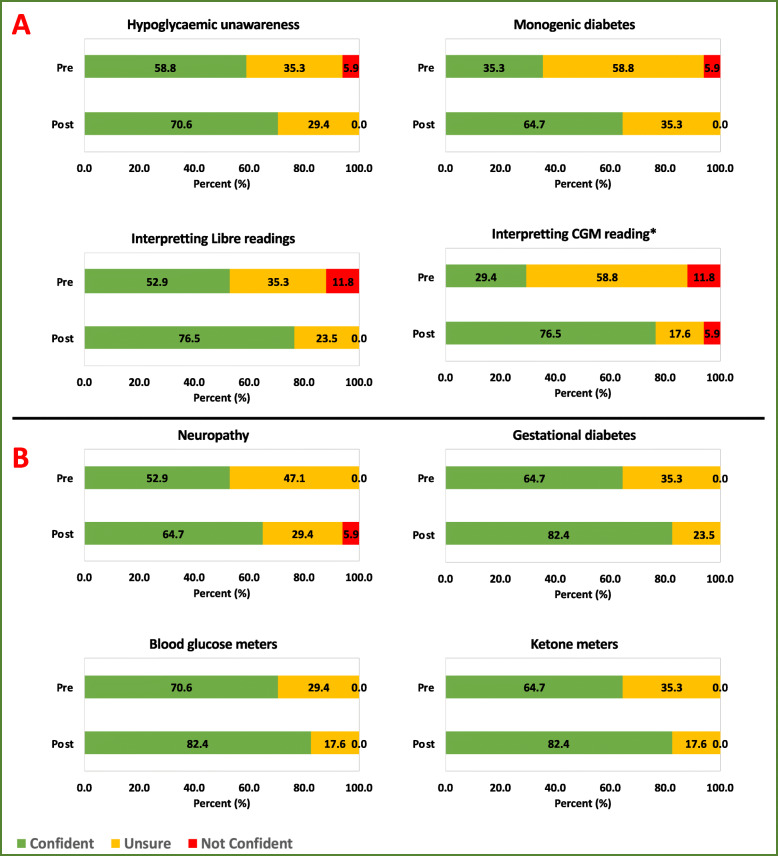
Illustration of changes in confidence levels for managing simulated (**a**) vs. non-simulated diabetes cases (**b**). *p < 0.05

In the diabetes session, there was a significant improvement in trainees’ confidence in interpreting CGM reading (*p* = 0.01). However, post-SIMBA change in self-reported confidence was not significant for interpreting Libre results (*p* = 0.1188), hypoglycaemic unawareness (*p* = 0.4207), and monogenic diabetes (*p* = 0.0744) (Fig. 4A). Similar to the endocrine session, we did not see any significant changes in trainees’ confidence for non-simulated cases; neuropathy (*p* = 0.6030), ketone meters (*p* = 0.2506), gestational diabetes (*p* = 0.2506), and blood glucose monitors (*p* = 0.4257) (Fig. 4B).

**Fig. 5:**
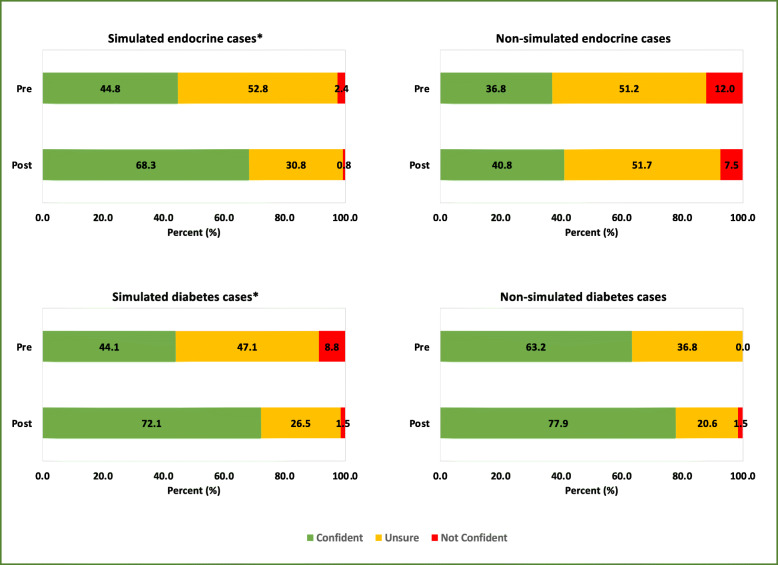
Illustration of changes in confidence levels for managing simulated vs. non-simulated cases. *p < 0.05

Overall, there was a significant improvement in trainees’ confidence in managing simulated diabetes cases (*p* = 0.0006) compared to non-simulated cases (*p* = 0.0713). (Fig. 5).

### Trainees’ feedback

In general, trainees reported they found the session interactive, practical, and relevant and they recommended it should be integrated into their regular training in the future. Some of the comments are quoted below:

“The simulation session was excellent. Very practical and relevant and led to good engagement and excellent discussion. Would definitely recommend continuing this and incorporating this into future training days”.

They were also happy with the chair contribution as they found the chair to be knowledgeable, interactive, and approachable.

“This has been the most useful session so far and would very much like to have more of these sessions. Very useful contribution from the chair”.

Despite all the positive feedback, we have also received some negative ones with regards to the interaction with moderators.

“Great, initially some lag but this was very minimal when more moderators joined. Excellent session”.

## Discussion

SIMBA is a social media/computer-based simulation where the case scenarios were extracted from real-life clinical experiences. Such cases not only helped mimic the clinical pathway but also the practical deviations needed to tailor to individual scenarios. Our moderators act as live patients and as a multi-disciplinary team (MDT) at different points of simulations but interact with the trainees through WhatsApp. The model was well received by the trainees as noted by their feedback. The use of an instant messaging platform (WhatsApp®) familiar to trainees, real-time interaction and specialist input could be the reasons for good reception.

We did not observe significant improvements in confidence levels for NFPA, macroprolactinoma, Cushing’s disease, and hypoglycaemic unawareness. NFPA and hypoglycaemic unawareness were included in the mock scenarios and hence the trainees may not see much change in their confidence whilst familiarizing themselves with the simulation model. The approach to Cushing’s disease has always been challenging even to experienced clinicians, therefore more simulation sessions might be needed for significant improvement in confidence.

To the best of our knowledge, this is the first-ever real-time simulation training in endocrinology and diabetes training using a common social medium. WhatsApp® has been previously used as an education tool in basic health sciences, clinical health sciences and medical education; it has been used in pathology to share images on WhatsApp®, to promote discussions of interesting cases, to share quiz questions and other related academic issues [8, 29]. There is also evidence to show WhatsApp® as an acceptable and practical way for teaching, connecting tutors from a range of specialities and across the wide geographical area to respective trainees and students [30]. In a recent review, Coleman et al. suggested WhatsApp® to be a suitable and effective teaching tool [31]. However, it is important to note that none of the studies included in this review has used WhatsApp® as a simulation model. Other social media platforms that have been used in this field include Facebook, Twitter and YouTube, but there is limited evidence showing that they improve performance outcomes—more studies are required to assess this [7, 9]. Furthermore, these social media were used to promote discussion and learning with information sharing [31], which is different from the model described here and hence not a good comparison.

Simulation-based learning in medicine is an emerging concept which is helping to move away from the apprentice-style “see one, do one, teach one” model. To the best of our knowledge, there is no universally accepted definition for medical simulation. In a recent systematic review for enhancing UK core medical training through simulation-based education, the Health Education England (HEE) quoted Professor David M Gaba from Stanford University stating “Simulation is a technique—not a technology—to replace or amplify real experiences with guided experiences that evoke or replicate substantial aspects of the real world in a fully interactive manner” [32, 33]. The HEE systematic review clearly identified the strength of simulation-based learning in emergency settings and practical procedures. However, we note from the review there is not sufficient simulation-based learning for elective care of which outpatient setting forms an important and integral part. The challenge for simulating elective care is its spread of care across months, if not years. SIMBA model takes this into account and compresses the patient journey into 30-min simulation experience. Therefore, we propose SIMBA as a simulation model for elective care and outpatient setting.

The greatest benefit of the SIMBA model is that it can be delivered with minimal resources. The social media platform is free, and the moderators and trainees were familiar with the model, requiring very little time to train. The only cost entailed for the whole model was for the venue charges to conduct the training. And there is scope to do away from needing a room as the session can be conducted virtually. Three of our moderators were based internationally (one in Georgia and two in Malaysia), as a proof of concept to having a virtual SIMBA Model. Also, the moderators do not need clinical expertise for this model but only familiarity with medical terminology. This provides the opportunity to recruit junior clinicians and/or medical students as moderators. We applied this in our diabetes session where 70% of our moderators were medical students. In return for their time and effort, the moderators gain valuable experience in managing the discussed clinical scenarios and in the field of medical education.

Trainees reported delays in replying through WhatsApp® in the endocrine session; we resolved this in the diabetes session with a better moderator to trainee ratio. We did not assess if there was a differential response depending on the level of clinical training in this study. There was also no control group to show that if the significant changes observed were due to our model independent of any confounders. We hope to include these and gather feedback from moderators in future studies to help further improve the model.

The model’s design is currently set to complete five cases in one sitting. The first case is a mock case for the trainee to familiarise with the simulation setting. This is followed by four cases in continuum after which these four cases were discussed in detail by an expert in an interactive setting. However, the future evolution of the model may have scope for trainees to dip in and out of various scenarios based on their availability. We are currently planning future studies which will include more baseline demographic information of the trainees (e.g.: level of training) to find out if there is a subgroup that will benefit more from the simulation (i.e. did the senior trainees perform better than the juniors). Dividing two groups of trainees into SIMBA and LBL with a higher sample size would provide us with more evidence for the use of this novel SBL model. Future sessions will also include a variety of other conditions in diabetes and endocrinology to assess the model’s strength across the speciality. If these studies can prove that SIMBA is an effective teaching model, this could be used in the future beyond this speciality. More studies will be needed to assess if this model also has an impact on trainee’s clinical practice.

## Conclusion

SIMBA proved to be an effective teaching model for pituitary conditions and to improve doctors’ confidence to interpret continuous glucose monitor reading and general management of complex diabetes cases. More studies are needed to further assess the effectiveness of this model in other endocrine conditions.

## Data Availability

All data generated or analysed during this study are included in this published article.
